# Sorption Speciation of Lanthanides/Actinides on Minerals by TRLFS, EXAFS and DFT Studies: A Review 

**DOI:** 10.3390/molecules15118431

**Published:** 2010-11-17

**Authors:** Xiaoli Tan, Ming Fang, Xiangke Wang

**Affiliations:** 1 Key Laboratory of Novel Thin Film Solar Cells, Institute of Plasma Physics, Chinese Academy of Sciences, P.O.Box 1126, Hefei 230031, Anhui, China; 2 Institute of Solid States Physics, Chinese Academy of Sciences, P.O. Box 1129, Hefei 230031, Anhui, China

**Keywords:** Sorption, Radionuclides, TRLFS, EXAFS, DFT

## Abstract

Lanthanides/actinides sorption speciation on minerals and oxides by means of time resolved laser fluorescence spectroscopy (TRLFS), extended X-ray absorption fine structure spectroscopy (EXAFS) and density functional theory (DFT) is reviewed in the field of nuclear disposal safety research. The theoretical aspects of the methods are concisely presented. Examples of recent research results of lanthanide/actinide speciation and local atomic structures using TRLFS, EXAFS and DFT are discussed. The interaction of lanthanides/actinides with oxides and minerals as well as their uptake are also of common interest in radionuclide chemistry. Especially the sorption and inclusion of radionuclides into several minerals lead to an improvement in knowledge of minor components in solids. In the solid-liquid interface, the speciation and local atomic structures of Eu(III), Cm(III), U(VI), and Np(IV/VI) in several natural and synthetic minerals and oxides are also reviewed and discussed. The review is important to understand the physicochemical behavior of lanthanides/actinides at a molecular level in the natural environment.

## 1. Introduction

For the long-term performance assessment of nuclear waste repositories, knowledge on the interactions of lanthanide/actinide ions with mineral surfaces is imperative. The mobility of released radionuclides is strongly dependent on the sorption and desorption processes occurring at mineral surfaces. Therefore, it is necessary to characterize the surface species formed and to elucidate the reaction mechanisms involved. An insight into the sorption mechanisms given by identification of surface species is of paramount importance for any predictive modeling of radionuclide migration, and the bioavailability of radionuclides in the natural environment [1,2].

Most studies of radionuclide uptake in mineral systems have been performed via macroscopic approaches (e.g., batch studies). The focus of these studies has been put on the determination of distribution coefficients, the use of adsorption isotherms, empirical and semiempirical equations (e.g., Freundlich, Langmuir, etc), and surface complexation models (e.g., constant capacitance, triple layer *etc*) to describe the sorption reactions of radionuclides at the solid-liquid interfaces. For example, the uptake of Eu(III) on oxides and clay minerals has been extensively investigated in our laboratory [3,4,5,6,7]. Accurate description of the sorption process is critical for environmental risk assessments and the development of sound remedial technologies in contaminant management. Therefore, a “mechanistic” surface complexation model was developed to predict the physicochemical behavior of radionuclides in the natural environment [8,9,10]. However, the surface complexation model assumptions with regard to sorption mechanisms and surface speciation cannot be directly derived from wet chemistry data. Equilibrium-based models describe the macroscopic sorption data simply and do not definitively prove a reaction mechanism [9,10]. Spectroscopic analysis can help to increase the reliability of model predictions by examining sorption mechanisms, surface speciation and local atomic structures at molecular level. To confirm the main sorption sites, it is essential to identify binding sites using spectroscopic techniques. 

Many different methods are useful to provide the microscopic information that is mandatory for making macroscopic predictions. In addition to conventional techniques such as resonance (nuclear magnetic resonance, electron spin resonance) and optical spectroscopy techniques (such as IR, UV-visible), electron microscopy and analytical chemistry, modern highly sensitive methods are needed. They are also either laser-based methods (photoacoustic spectroscopy, thermal lensing spectroscopy, time-resolved laser fluorescence spectroscopy) or synchrotron-based X-ray absorption spectroscopy (XAS). Among the various techniques available, time resolved laser fluorescence spectroscopy (TRLFS) and extended X-ray absorption fine structure spectroscopy (EXAFS) are applicable to radionuclide-mineral systems because of their high sensitivity and selectivity to targeted ions [11,12,13,14]. These methods were largely used to determine the speciation and local atomic structures of radionuclides in aqueous and non-aqueous solutions, at the mineral/water interface, or incorporated into the bulk mineral phase. Regarding these two methods, the application of TRLFS is confined to determine the coordination chemistry of Eu(III), Tb(III), and some actinide ions [15,16]. While EXAFS was employed to determine the structural parameters of actinide/lanthanide ions adsorbed on mineral surfaces at molecular level. Inner-sphere *versus* outer-sphere complexation, mononuclear *versus* multinuclear, or solid solution formation can be distinguished through a local atomic structure analysis of EXAFS and TRLFS.

In addition to the increasing importance and the remarkable improvement of the experimental surface methods, theoretical calculations are also becoming routinely used as an indispensable tool in chemical research. As one of the most useful theoretical tools, density functional theory (DFT) arises mainly because of its ability of accounting for the correlation energy (important in the treatment of metal systems) in a very efficient way [17], which is usually applied to describe the adsorption of molecules on a surface as a localized interaction. DFT is used to give an insight into adsorption processes at the atomic level and to contribute to the interpretation of experimental results from X-ray photoelectron spectroscopy (XPS), TRLFS and EXAFS *etc.* [18,19,20].

In this paper we review the utilization of TRLFS, EXAFS and DFT to characterize the speciation and local atomic structures of radionuclides adsorbed on minerals and oxides. A number of groups are working worldwide in this field. This review will not cover all published results but will rather focus on a few notable examples that may enlarge the people view on the sorption species and *in-situ* structures of actinides/lanthanides on mineral and/or oxide surfaces. It also made a comparative investigation of radionuclide sorption onto different solid surfaces by EXAFS and TRLFS techniques. The DFT is directly correlated to the experimental outcome in order to better understand sorption processes of radionuclides on mineral surfaces. In addition, based on the EXAFS and TRLFS analysis, the contributions of humic substances and carboxylate sites, possible binding sites of actinides/lanthanides on different mineral and oxide surfaces, at various experimental conditions (such as pH, ionic strength *etc*.) are also reviewed and discussed.

## 2. Time Resolved Laser Fluorescence Spectroscopy (TRLFS)

The TRLFS technique enables the speciation of lanthanides like Eu(III) and actinides like U(VI), Am(III) and Cm(III) in aqueous solutions and on the water/mineral interfaces. TRLFS provides two types of information: (1) changes in the emission spectra are due to variations in the first coordination sphere of the fluorescent metal ion and indicate inner-sphere complexation processes; (2) analysis of the fluorescence lifetime allows one to determine the number of fluorescence quenching entities present [21]. 

The major advantages of TRLFS over other techniques, such as EXAFS and NMR, are its enhanced sensitivity and its combined information on concentrations (based on intensities) and coordination (based on emission wave numbers and fluorescence lifetimes) [11,22]. TRLFS also has the benefit that it is a noninvasive and *in-situ* method for the direct investigation of solutions, solids, and adsorbates. A drawback of this method is the limited number of species of interest that are fluorescent (such as uranium), and the measurements are dependent on temperature and strongly influenced by apparatus properties [11]. Thus, TRLFS is not a universal method.

Many researchers have studied the speciation of fluorescent lanthanides/actinides or their complexation with organic [e.g., humic acid (HA), fulvic acid (FA), citric acid] and inorganic ligands (e.g., carbonate, sulfate, phosphate) [12,23,24,25,26,27,28,29,30]. Recently the application of this method was extended to study the characteristics of adsorbed lanthanides/actinides on solid materials [11].

In this section the study of lanthanide/actinide speciation on a molecular scale by means of TRLFS is presented. We first begin with the relationship between fluorescence lifetimes and the hydration water molecules in the first coordination shell of lanthanides/actinides in aquatic environment. In the remaining portion selected examples of lanthanide/actinide sorption speciation by means of TRLFS are presented. A short discussion of the spectroscopic characteristics, including the physical origin of the select lanthanide/actinide spectra and experimental aspects are described and reviewed. A summary of pedagogic aspects meant for the reader to become acquainted within this review is in Section 2 and is intended as a self-check. 

### 2.1. Lifetime and the Hydration Water Molecules

Fluorescence lifetimes of lanthanides/actinides in aquatic environment are relatively short due to the energy transfer from excited f levels to lower lying vibronic states of water molecules in first coordination sphere of the lanthanides/actinides. When the lanthanide/actinide ions are adsorbed onto a mineral surface by inner-sphere complexation, some of the H_2_O molecules in the first coordination sphere are displaced, and thereby results in extended fluorescence lifetimes. The fluorescence lifetime provides an insight into the chemical environment of Eu(III), Cm(III) and Am(III) complexes in solution or adsorbed on solid phases. The fluorescence lifetimes (τ) of the species are calculated from an exponential decay function, which has the following form for the species:

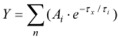
(1)
where *Y* is the measured fluorescence intensity at the time *x*, *A_i_* is the fluorescence intensity of the *i-*th species at the time 0, and *τ_i _* is the fluorescence lifetime of *i-*th species. The fluorescence lifetime values of Eu(III), Cm(III) and Am(III) are particularly interesting as they are linked to the number of hydration water molecules, 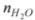, in the first coordination sphere of Eu(III), Cm(III) and Am(III). The value of 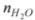 was calculated from the lifetime, *τ* (*k_obs_* decay constants (ms^-1^), 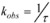), using the empirical law (equations 2-4) [15,16]: 


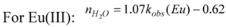
(2)


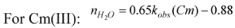
(3)


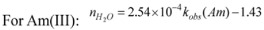
(4)

### 2.2. Eu(III) TRLFS Study

Eu(III) was used as an analogue of the trivalent lanthanides/actinides in wet chemistry studies. On the basis of the similarities in the complexation behavior of trivalent lanthanides and actinides with comparable ionic radius [31,32], it is anticipated that Eu(III) is a suitable analogue for Cm(III). 

#### 2.2.1. Spectroscopic characteristics of Eu(III)

**Figure 1:**
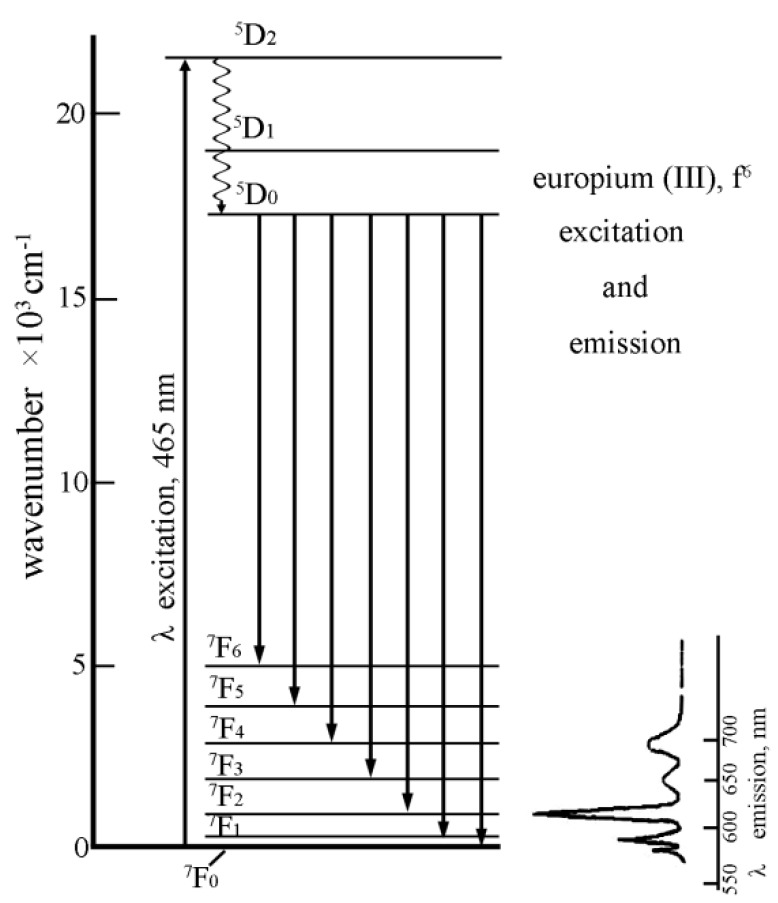
Electronic energy scheme for Eu(III) [15].

The Eu(III) complexes were excited to the ^5^D_2_ state by a laser flash which resulted principally in emission from the ^5^D_0_ excited state as depicted in the energy level diagram of Figure 1. Laser-induced excitation of the state is followed by rapid (< 5 µs**) **nonradiative relaxation (wavy line) to the luminescent ^5^D_0 _state. Emission to the ground ^7^F manifold from the ^5^D_0_ state is indicated by the heavy arrows with a “typical” intensity profile of the emission bands depicted in the figure to the right.

**Figure 2:**
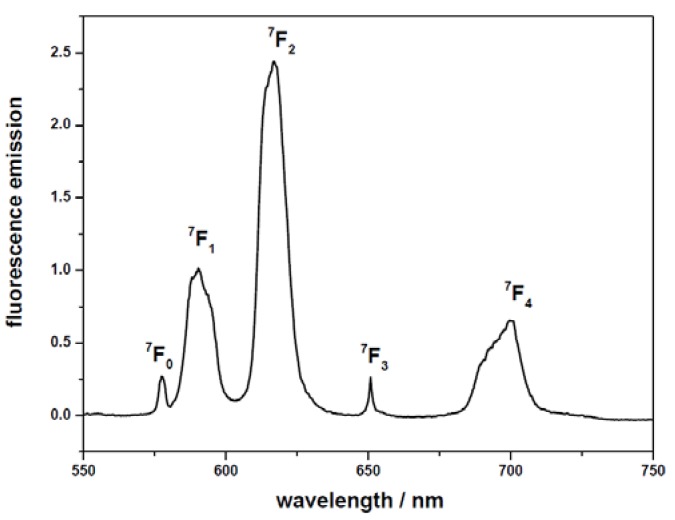
Eu^3+^ fluorescence spectroscopy in aqueous solution. Different peaks represent different electron transition. [Eu] = 4.1 × 10^-6 ^mol/L, pH = 4.0 ± 0.1, T = 20 ± 1 ºC.

Herein only TRLFS measurements with non-selective excitation (394 nm) are discussed. Direct excitation of the ^5^D_0_ level is often used to get more precise information of the Eu(III) species which are involved. Figure 2 shows the “typical” fluorescence spectroscopy of Eu^3+^ ions in aqueous solution. Eu(III) fluorescence spectroscopy is characterized by five electron transitions as follows: ^5^D_0_→^7^F_0_ (λ = 579 nm), ^5^D_0_→^7^F_1_ (λ = 594 nm),^ 5^D_0_→^7^F_2 _(λ = 619 nm),^ 5^D_0_→^7^F_3_ (λ = 651 nm),^ 5^D_0_→^7^F_4_ (λ = 702 nm). The positions of the Eu(III) luminescence bands (^5^D_0_→^7^F_1-4_ transitions) are almost independent of the chemical environment of the Eu(III) ion [1]. Only the intensity for the ^5^D_0_→^7^F_2_ transition, changes significantly on variation of water molecules in the first coordination sphere due to the so-called “hypersensitive effect” [1,33]. The ratio of the emission intensities obtained for the ^5^D_0_→7F_1 _(λ = 592 nm) and ^5^D_0_→^7^F_2 _(λ = 617 nm) transitions together with the fluoresence lifetime provide valuable information on the Eu(III) speciation, which is quite important to understand the sorption mechanism and species of Eu(III) in aqueous solutions on adsorbed on solid particles [34]. The experimental fluorescence lifetime for the Eu^3+ ^aquo ion is 110 ± 5 µs in water [15,21,33,34]. A linear correlation is observed between the decay rate and the number of H_2_O molecules in the first coordination sphere of Eu(III) (equation 2), where a lifetime of 110 µs corresponds to 9 and 1725 µs corresponds to zero H_2_O molecules in the first coordination sphere [15]. Additional information can be derived by obversation from the ^5^D_0_→^7^F_0_ transition [35]. 

#### 2.2.2. Eu(III) adsorbed on minerals

**Figure 3:**
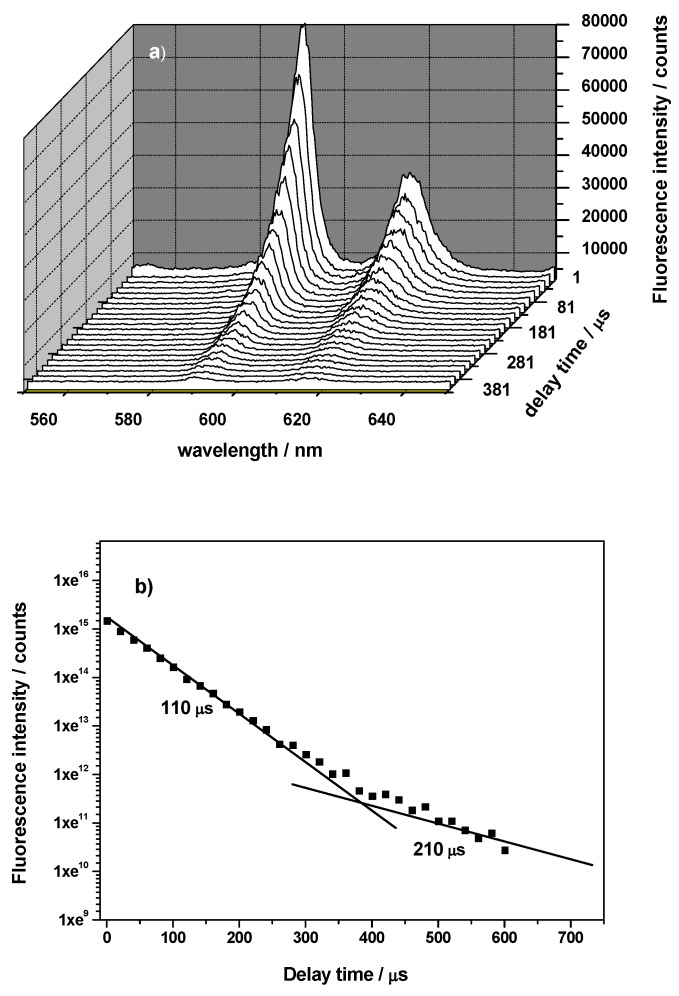
Three-dimensional spectra (a) and time dependent fluorescence spectra (b) of Eu(III) in alumina suspension. [Eu(III)] = 4.1 × 10^-6^ mol/L, m/v = 0.44 g/L, T = 20 ºC, pH = 5.3 ± 0.1, I = 0.1 mol/L NaClO_4_.

The surface sorption process of Eu(III) onto oxides and clay minerals has also been investigated by TRLFS [1,21,32,33,34,35,36]. Figure 3 shows three-dimensional spectra and time dependent fluorescence spectra of Eu(III) in alumina suspension. The intensity of the peak decreases with increasing the delay time. The obtained emission decay of the Eu-Al_2_O_3_ species is bioexponential, and the lifetime is found to be 110 and 210 μs (210 μs, which corresponds to five water molecules in the first Eu(III) coordination shell), which indicates that there are two Eu(III) species under this condition. The results of TRLFS analysis suggest that at low pH the metal ion keeps its complete hydration sphere indicating unsorbed free Eu^3+^ ions in aqueous solutions. The number of H_2_O molecules in the first coordination sphere decreases from nine to five, indicating the formation of inner-sphere surface complexes [1]. 

At high pH, the change of the (transition) ratio of the fluorescence emission of Eu(III) with 2~3 H_2_O molecules indicates that the inner-sphere surface complex with the surface hydroxyl groups or direct sorption of Eu(III) inorganic complexes (carbonated or hydroxide) were formed [34]. In order to characterize the Eu(III)/clay surface complexes in the persence and absence of carbonate, Stumpf *et al*. [33] carried out the experiments under atmospheric conditions and in carbonate free atmosphere conditions. They found that when carbonate was excluded the lifetime of the surface adsorbed Eu(III) species was 188 ± 20 μs. No change of the intensity ratios for ^7^F_1_/^7^F_2_ is detected when Eu is completely adsorbed or speciation is changing by further increase of pH and formation of ternary hydrolyzed surface complexes. While under atmosphere conditions, the increase in intensity of the ^7^F_2_ transition and increase of lifetime were explained by the formation of ternary clay/Eu(III)/carbonate complexes. TRLFS with Eu(III) allows us to distinguish between surface sorption and incorporated onto the lattice of a given mineral. Stumpf *et al*. [35] reported in the Eu(III) doped Mg-Al-Cl-hydrotalcite system, two different Eu(III)/hydrotalcite species were identified by TRLFS measurements at low temperature. Emission spectra of the ^5^D_0_→^7^F_0_ transition and the lifetime of 305 ± 5 µs suggest that Eu(III) ions are corporated into the hydrotalcite lattice. By comparison with the TRLFS results obtained from the Eu/alumina and Eu/silica systems, Kowal-Fouchard *et al*. [36] reported that in the Eu(III)/montmorillonite system, the lifetime of 250 µs corresponds to Eu(III) sorption onto “aluminol” edge sites while the lifetime of 135 µs corresponds to its retention by “silanol” edge sites of the montmorillonite. They proposed “a” Eu(III) sorption onto Na- montmorillonite with three different clay sites: exchange sites, “aluminol” and “silanol” edge sites, depending on the pH value and the ionic strength of the suspension. From the TRLFS analysis, one can see that the species of Eu(III) adsorbed on the different sites of minerals or oxides can be identified, and thereby the sorption mechanism of Eu(III) at different conditions can be evaluated from the adsorbed species. 

#### 2.2.3. The interaction of Eu(III) with HS or HS-mineral hybrids

**Figure 4:**
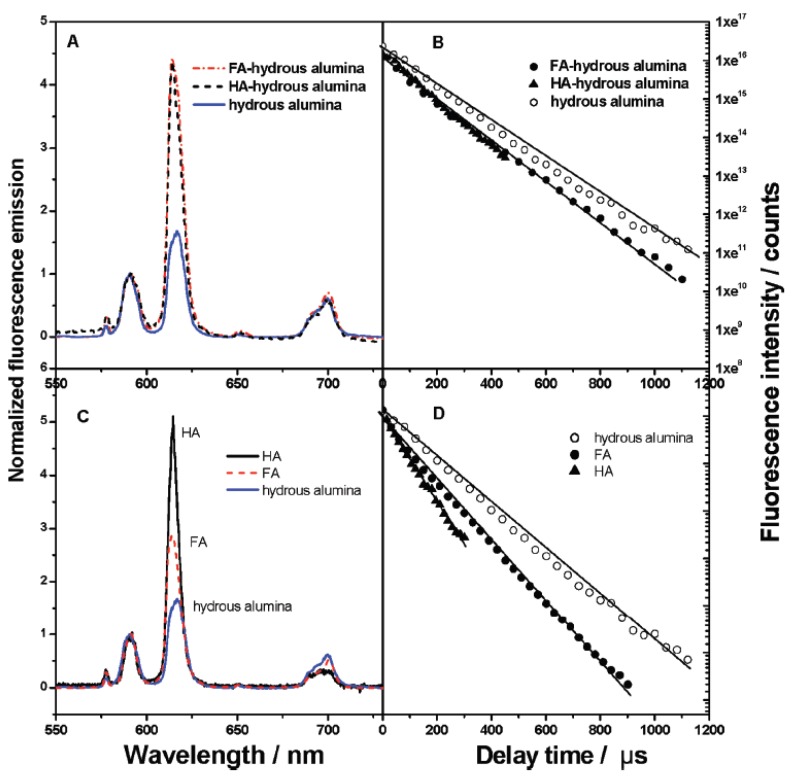
TRLFS results for Eu(III) bound to HA, FA, hydrous alumina, HA-hydrous alumina, and FA-hydrous alumina hybrids. C(hydrous alumina) = 4.4 g/L, C(Eu(III))_(initial)_ = 4.3 × 10^-5^ mol/L, C(FA/HA)_(initial)_ = 10 mg/L, C(KNO_3_) = 0.1 mol/L, pH = 6.3 ± 0.1, T = 20 ± 1 ºC [37].

Humic substances (HSs) account for a significant fraction of natural organic carbons in surface waters and soils, and play many important roles as a complexing agent for Eu(III) ions, and the adsorbed HSs on mineral surfaces affects the mobility and bioavailability of aquatic Eu(III) ions in the natural environment because of the strong complexation ability of HSs with Eu(III) ions by the formation of soluble Eu-HS complexes with free HSs in solution or the formation of Eu-HS on solid surfaces with surface adsorbed HSs. The nature of the interaction between lanthanide/actinide ions and HSs depends on the chemical state of the organic material, which can be soluble or associates with mineral particles. The interactions of Eu(III) with HSs or HS-mineral hybrids have been characterized structurally at the molecular level by many spectroscopy techniques [23,24,37,38,39]. A comparison of TRLFS spectra shows clear differences of Eu(III) with free soluble HA or FA in solution and with surface adsorbed HA and FA on alumina surfaces (Figure 4). The relative emission intensities of Eu(III) at the wavelength of 619 nm in Al_2_O_3_, fulvic acid (FA) and humic acid (HA) solutions are quite different. Due to the quenching properties of HA/FA, it is not accurate to calculate the fluorescence lifetime of Eu(III) in the presence of HA/FA. However, the different fluorescence lifetimes are still interesting because it indicates that different species of Eu(III) is formed in the presence of HA/FA [37]. The interaction of Eu(III) with mineral-adsorbed organic substances is also different from that with dissolved HSs. The species of Eu(III) in ternary organic-inorganic hybrids are as a Eu(III)-HS complex on mineral surface [37]. Montavon *et al*. [38] also detected one species in the Eu/PAA/Al_2_O_3_ ternary system with three water molecules in the first coordination shell. The results are in accordance with the literature that the interaction of Eu(III) with one COO- group leads to an expulsion of two water molecules and the formation of monodentate surface sites between Eu(III) and the aluminol group lead to the loss of four water molecules [40,41]. At low metal ion concentration, the appearance of another lifetime with one water molecule in the first coordination shell indicates that the metal ion is no longer adsorbed at the surface, but a possible migration of Eu(III) bound to surface sites into the adsorbed organic layer in the inner sphere. Takahashi *et al*. [42] studied the effect of HA on the sorption of Eu(III) on kaolinite and silica, and found that the speciation of Eu(III) was affected by different addition sequences of Eu(III) and HA. In our laboratory, we also studied Eu(III) sorption on Al_2_O_3 _by batch and spectroscopy techniques, and the TRLFS results showed clearly that the species of Eu(III) adsorbed on HA-Al_2_O_3_ hybrids are affected by the addition sequences of Eu(III) and HA although the amount of Eu(III) adsorbed on HA-Al_2_O_3_ hybrids are not influenced by the addition sequences from the batch experiments.

### 2.3. Cm(III) TRLFS Study

In applications of fluorescence properties in speciation studies of actinide(III) and lanthanide(III) ions, Cm(III) is preferable to Eu(III) since the detection limit for Cm(III) is much lower than that for Eu(III) by two orders of magnitude, allowing the use of lower concentrations of Cm(III) than Eu(III) [43]. 

#### 2.3.1. Spectroscopic characteristics of Cm(III)

**Figure 5:**
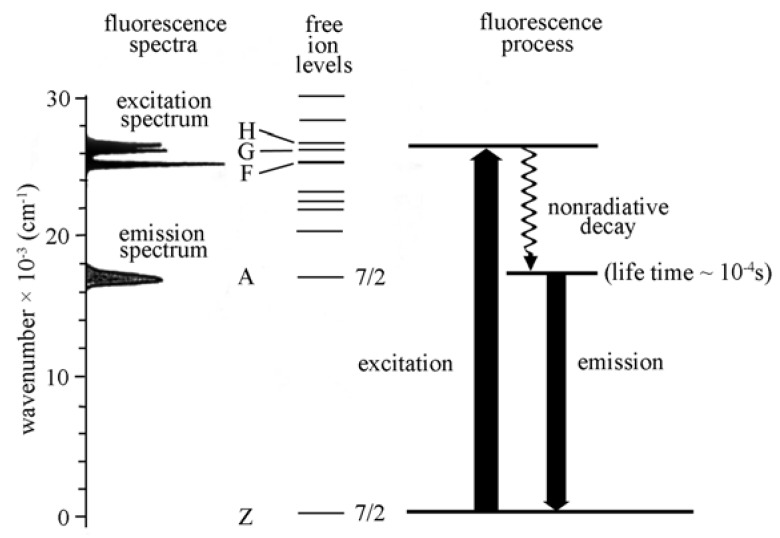
Free ion energy levels of Cm^3+^ ions and spectroscopic characteristics [26].

The energy scheme of the free Cm^3+^ ion in aqueous solution is illustrated in Figure 5. The absorption spectroscopy of Cm^3+^ is characterized by three strong f-f transitions from the Z-ground state (^8^S_7/2_) to excited states G, H, and F. A subsequent deexcitation undergoes a nonradiative decay to the A-state (^6^D_7/2_), from which a significant radiative decay occurs down to the ground state with a relatively high quantum efficiency yielding an emission at 593.8 nm [26]. Edelstein *et al*. [44] reviewed the fundamental electronic structure and spectroscopy of the Cm(III) ion in crystals and then described how the fluorescence spectra and fluorescence lifetimes of the Cm(III) in solutions or on surfaces can be utilized to investigate the coordination geometry.

In contrast to the Eu(III) spectroscopy, the Cm(III) ^6^D_7/2_→^8^S_7/2_ transition energy is influenced by inner-sphere complexation and the emission band of the free aquo ion (λ = 593.8 nm) is shifted to higher wavelengths. The lifetime of the radiative emission process is determined to be τ = 63 ± 3 µs in HClO_4_ solution for the hydrated Cm^3+^ ions [26].

#### 2.3.2. Cm(III) interaction with minerals

Up to now, the interactions of Cm(III) with mineral surfaces like silica [45], aluminum oxides or hydroxides [46,47,48,49], clay minerals [41,43,50], CSH phases [51], cement [52], feldspar [53], calcite [54,55], and α-alumina single crystal surfaces [56] have been investigated. Moll *et al*. [57] studied the interaction of Cm(III) with microbes. Others also investigated the influence of humic substances and dissolved CO_2_ on the sorption of Cm(III) on mineral surface [43,58].

**Figure 6:**
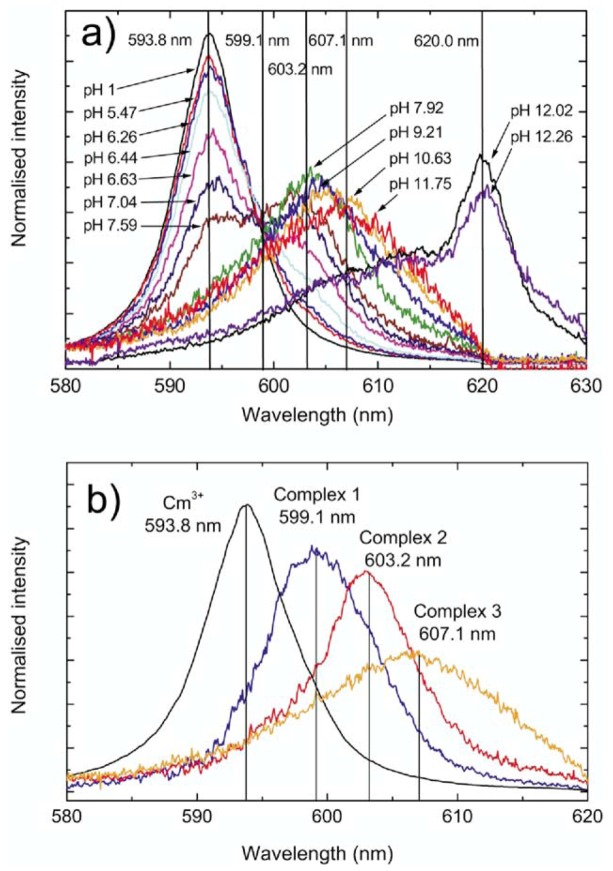
Cm-fluorescence spectra at different pH values in the presence of Ca-montmorillonite. (a) Cm fluorescence spectra normalized to same peak area. Total Cm concentration = 2.5 × 10^-7^ mol/L, m/V ratio = 0.25 g/L, 0.066 mol/L Ca(ClO_4_)_2_ solution. (b) Spectra of individual surface complex species derived from peak deconvolution. Surface precipitated species (λ = 620 nm) are not considered in the peak deconvolution [21].

The normalized Cm-fluorescence spectra at different pH values in the presence of Ca-montmorillonite are demonstrated in Figure 6. With increasing pH, the intensity of Cm^3+^ aquo ion peak decreases and leads to a red-shift of the fluorescence emission. The red shifted band can be ascribed to curium species adsorbed onto the montmorillonite surface. A peak deconvolution was carried out to resolve the individual species from the composite fluorescence emission spectra at pH > 5. Five different species have been identified. In addition to the Cm(III) aquo ion, three different emissions corresponding to the inner-sphere Cm(III) surface complexes are founded at 599.1, 603.2 and 607.1 nm. At pH ≥ 12, a dramatic shift of the 620 nm emission band suggests that the Cm is incorporated into a surface precipitate [21].

TRLFS spectra show that only the peak at 293.8 nm is detected at low pH value. In general outer-sphere complexation cannot be differentiated from the aquo ions based on the fluorescence spectra. This observation indicates that the Cm(III) is not adsorbed or the formation of outer-sphere surface complexes on the interlayer sites. The formation of inner-sphere surface complexes varying with pH was observed. At high pH Cm(III) may incorporate into a surface precipitate at the mineral surface. Researchers reported that the value of 115-120 µs for the fluorescence lifetime of Cm(III) sorption to mineral surfaces such as clay minerals [21,41,59], quartz [60] and Al_2_O_3_ [46,49], and the coordination with 5 H_2_O/OH^-^ as characteristic for Cm(III) species adsorbed onto a number of mineral surfaces by inner-sphere surface complexation. Measured fluorescence lifetimes of adsorbed Cm(III) and peak deconvolution of Cm-spectra are consistent with the formation of surface complexes of ≡S-O-Cm(OH)_x_^(2-x)^(H_2_O)_5-x_ [21]. 

**Table 1:** TRLFS characterization of Cm(III) species at Al_2_O_3_ and clay mineral surfaces.

System	pH	Peak(nm)	τ(µs)	n(H_2_O)	Possible complexes	Reference
γ-Al_2_O_3_	4.40-	601.2	110	5	≡Al-O-Cm^2+^(H_2_O)_5_	[46]
	9.56	603.3	110	5	≡Al-O-Cm^+^(OH)(H_2_O)_4_	
γ-Al_2_O_3_	4.92-	593.8	65	9	Cm^3+^(H_2_O)_9_	[49]
	13.16	600.6	110	5		
		602.5	110	5	≡Al-O-Cm^+^(OH)_x_^(2-x)^(H_2_O)^5-x^	
		605.7	110	5		
α-Al_2_O_3_(single crystal)						
(001)	4.5	601.3	107±1	5.0		[55]
(012)	and	603.2	162±3	3.1		
(110)	5.1	603.5	192±3	2.5		
(104)		603.8	185±3	2.6		
(018)		603.6	158±3	3.2		
Ca-Montmorillonite	5.47-	599.1	120±15	4.5		[21]
	12.26	603.2	120±15	4.5	≡S-O-Cm(OH)^(2-x)^_x_(H_2_O)_5-x_	
		607.1	120±15	4.5		
		620	753±88	0	surface precipitate	
Na-illite	4.68-	598.8	115±4	4.8		[21]
	12.02	602.3	115±4	4.8	≡S-O-Cm(OH)^(2-x)^_x_(H_2_O)_5-x_	
		605.5	115±4	4.8		
Kaolinite	3.6-	593.8	68±3	9.0	Cm^3+^(H_2_O)_9_	[41]
smectite	8.3	598.8	110±7	5.0	≡Al-O-Cm^2+^ (H_2_O)_5_	
		603.3	110±7	5.0	≡Al-O-Cm^+^ (OH)(H_2_O)_4_ or	
					≡(Al-O)_2_-Cm^+^ (H_2_O)_5_	
quartz	3.75-	593.8	68±3	9.0		[60]
	9.45	601.4	123±10	4.4		
		603.6	123±10	4.4		
Silica	4.93-	593.8	68±3	9.0	Cm^3+^	[45]
	9.19	602.3	220±14	2.1	≡SiOCm(I)	
		604.9	740±35	0	≡SiOCm(II)	

Stumpf and Rabung’s [41,46,49,52,53,54,55,56,60] group at the Karlsruhe Research Center (Germany) carried out Cm(III) sorption studies on different mineral surfaces using TRLFS technique. They observed clear similarities in the TRLFS for Cm(III) adsorbed on clay minerals, γ-Al_2_O_3_ and α-Al_2_O_3_(001) (see Table 1). Similar positions of the fluorescence emission bands indicated a very comparable chemical environment of mineral bound Cm(III) ions. They therefore concluded that in all cases the Cm(III) ion binds to the same type of aluminol groups [59]. Evaluation of fluorescence lifetimes suggests the formation of ≡Al-O-Cm(OH)_x_^(2−x)^(H_2_O)_5-x_ inner-sphere surface complexes. They also studied incorporation of Cm(III) in siliceous [60] and calcite [55], and similar results were also reported. The species of Cm(III) on solid particles are quite similar at similar experimental conditions, which may imply that the sorption mechanism of Cm(III) is relatively dependent on the environmental conditions.

**Figure 7:**
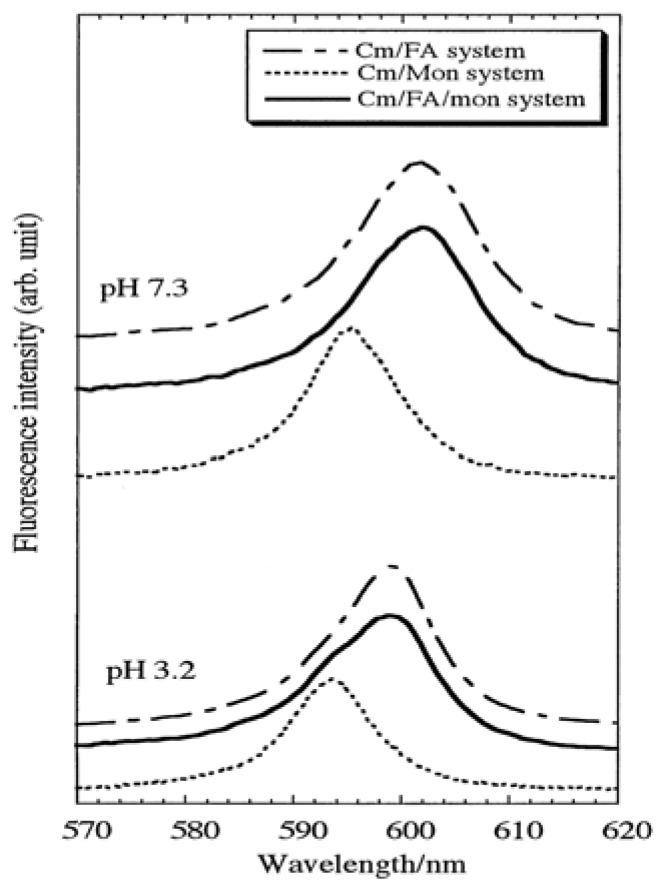
Fluorescence spectra of Cm(III) species in three systems: Cm(III)-fulvate (Cm/FA), Cm(III) species adsorbed on montmorillonite (Cm/mont), and Cm(III) species adsorbed on FA-montmorillonite hybrid (Cm/FA/mont). Total concentrations of Cm(III), fulvic acid, and montmorillonite are 2.0 × 10^-6^ mol/L, 100 mg/L, and 4.0 g/L, respectively [43].

TRLFS is also useful for the speciation of Cm(III) complexes with humic substance and model ligands [25,26,27]. Panak *et al*. [25] reported that the peak maximums of the Cm(III) humate and fulvate fluorescence emission spectra were shifted from 593.8 nm for the uncomplexed Cm^3+^ aquo ions to 601.0 and 600.3 nm, respectively. The number of water molecules in the first coordination shell is calculated to be 3 ± 1 for the Cm(III) humate/fulvate complexes, follows that Cm(III) forms a highly coordinated inner-sphere surface complexes with humic acid and fulvic acid. Takahashi *et al.* [43] characterized the Cm(III) adsorbed on fulvic acid-montmorillonite hybrids (organic-inorganic hybrids). They reported that in the absence of fulvic aicd at pH > 3.2, more than 80% of the Cm(III) was removed from aqueous solution due to the sorption on montmorillonite. In the presence of fulvic acid, less Cm(III) was adsorbed, due to the formation of dissolved Cm(III)-fulvic complexes with soluble fulvic acid that kept Cm(III) in the aqueous phase. The spectrum of the Cm/FA/montmorillonite system has a shoulder at the peak wavelength of the Cm/montmorillonite system (Figure 7), and its main peak coincides with that of the Cm/FA system, which indicates that the Cm(III) is adsorbed directly on montmorillonite, and Cm(III) is adsorbed as fulvate complexes coexist on the particulate matter. 

At pH 7.3, no contribution from inorganic Cm(III) species adsorbed on montmorillonite is detected and the Cm(III)-fulvate complex is the dominant Cm(III) species adsorbed on the FA-montmorillonite hybrids. The species of Cm(III) on mineral surfaces are not only dominated by the properties of mineral and the properties of humic substances, but also affected the pH values and other conditions such as ionic strength, temperature *etc.* because the interaction of humic substances with minerals is dominated by many different parameters. The systematic investigation of Cm(III) species on minerals is critical importance in future.

In the case of cation exchange reactions taking place at low pH (< 5), the actinide hydration sphere remains unchanged during outer-sphere surface complex formation. Hartmann *et al.* [61] used the TRLFS to differentiate between nonsorbed aquo ions and outer-sphere adsorbed Cm(III) onto different montmorillonites at pH 4.0~4.2. They also studied Cm(III)/clay outer-sphere surface complexation at different ionic strengths using NaCl as the background electrolyte. They detected that fluorescence lifetimes were reduced due to the introduction of iron in the clay. They found that with increasing Na^+^ concentration (> 0.1 mol/L) the proportion of the shorter fluorescence lifetime is reduced and the fluorescence lifetime of 65 μs of nonsorbed Cm^3+^ aquo ion is dominating, which means the proportion of adsorbed Cm^3+^ ions decreases. At an ionic strength of ~0.19 mol/L more than 99% of the actinide ions are in solution. The sorption and species of actinides is not only affected by the pH values as mentioned above, but also influenced by the ionic strength as the ionic strength can affect the surface properties of minerals and thereby can affect the sorption of actinides on minerals. 

#### 2.3.3. The quenching influence of iron

The iron incorporated in the bulk structure of the clay mineral causes fluorescence emission quenching. This quenching effect has been observed by many authors in their sorption studies using the TRLFS technique. The quenching effect of iron is due to a nonradiative energy transfer from the excited Cm(III) to Fe(III) by dipole-dipole interaction. The extent of the quenching effect is dependent on the iron concentration and the distance between the fluorescent Cm(III) and iron. It is reported that a part of Cm(III) is bound to ≡Fe-OH groups and leads to the formation of non-fluorescent surface complexes and the complete extinction of the fluorescence light [21,53]. The strong decrease in fluoresence intensity of the surface complexed Cm(III) at high pH is due to the enrichment of Fe at the surface [21]. Sorption of Cm(III) to amorphous FeOOH being present as an impurity in the clay minerals can also be, to a certain extent, responsible for the fluorescence intensity decrease. Through the quenching effect, Hartmann *et al*. [61] found that in the presence of Fe^3+^, the fluorescence emission intensity decreases as a result of a shorter fluoresence lifetime, and therefore a significant different between the fluoresence lifetimes between the outer-sphere adsorbed Cm(III) and Cm^3+^ aquo ions is observed, which is useful to evaluate the sorption species and mechanism of Cm(III) on mineral at different conditions.

### 2.4. U(VI) TRLFS Studies

Under environmental conditions, uranium typically occurs in the hexavalent form as the mobile aqueous uranyl ion (UO_2_^2+^). The U(VI) species can also be used as an analogue of actinide ions like Np(VI) or Pu(VI) [2,62]. The sorption of UO_2_^2+^ ions onto solid surfaces has been widely studied because this process has a significant effect on transport properties.

#### 2.4.1. Spectroscopic characteristics of U(VI)

**Figure 8:**
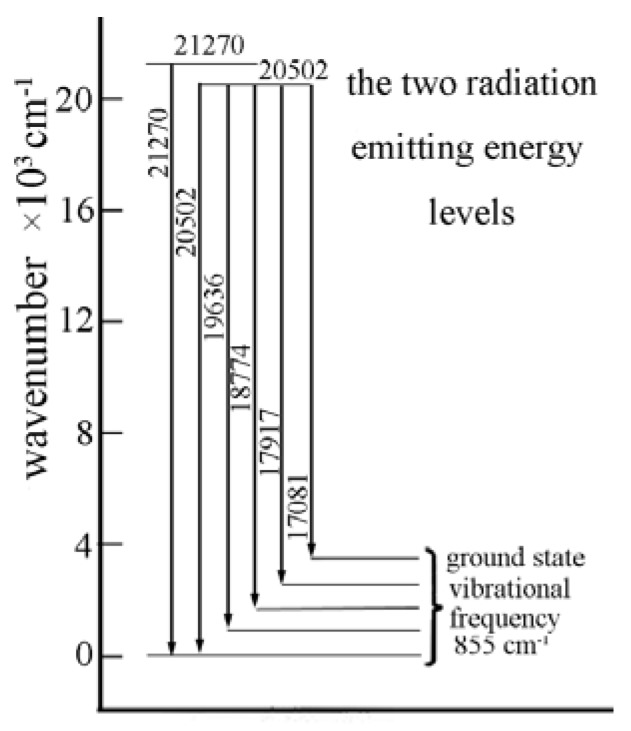
Transition energies of the aqueous uranyl ion in perchlorate medium as determined from the resolved emission spectra. Radiative emission to the ground term is indicated by the heavy arrows with a “typical” intensity profile of the emission bands (figure has been modified and taken from reference [62]).

**Figure 9:**
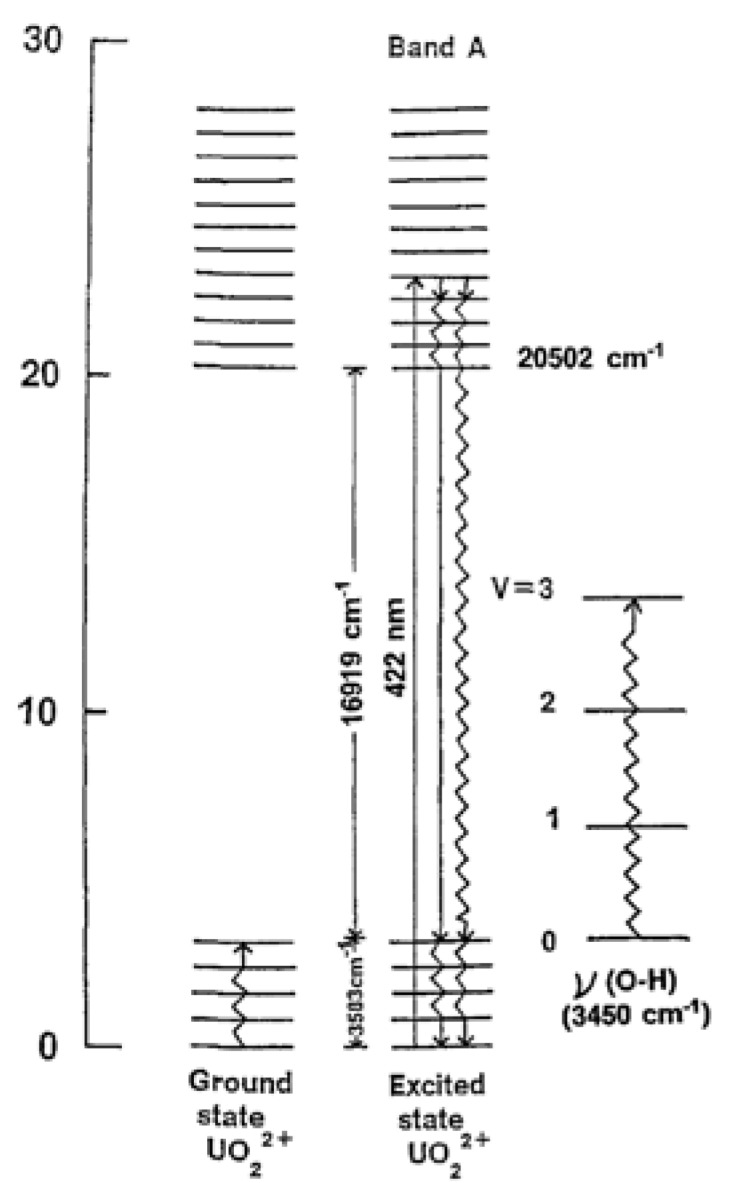
Inter- and intramolecular nonradiative energy transfer in solid UO_2_^2+^-*Datura* samples [63].

The positions of the fluorescence emission bands are intrinsic property of the U(VI) TRLFS spectrum, whereas the fluorescence lifetime is more dependent on the sample preparation (wet or dry) and temperature of the experiment [62]. The fluorescence emission spectrum of UO_2_^2+^ is mainly originated from the electron transitions from two emission levels at 21,270 and 20,502 cm^-1^ to five vibrational levels in the ground state (Figure 8). The five lowest-energy fluorescence emission bands have an average spacing of 855 cm^-1^[62]. A schematic diagram of the situation for the UO_2_^2+^ is also shown in Figure 9.

**Figure 10:**
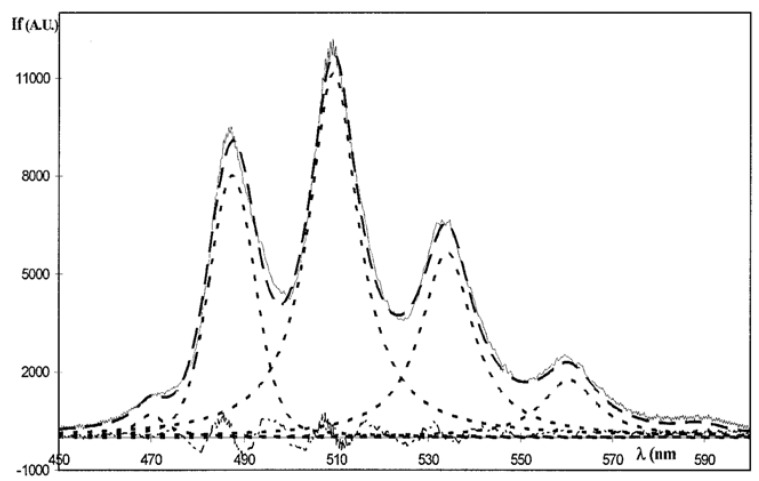
UO_2_^2+^ (free uranyl ions) fluorescence spectrum together with convoluted spectrum. [U] = 4.0×10^-8^ mol/L, I = 0.1, pH = 1 [64].

The deconvoluted free uranyl ions fluorescence spectrum has six characteristic peaks located at around 470, 488, 509, 533, 559, and 585 nm [64,65]. The four main peaks (488, 509, 533, and 559 nm) have an average full width at mid-height (FWMH) of around 13 nm (Figure 10). Fluorescence lifetime is between 1 and 2 µs. UO_2_^2+^, free uranyl, is taken as a reference in terms of spectroscopic scale since it is very likely that all the other species present a bathochromic shift due to complexation and a broadening due to the addition of new vibrational modes.

#### 2.4.2. Uranium-hydroxo complexes

The TRLFS technique was applied to make a rather speculative characterization of the different uranium-hydroxo complexes [64,65,66]. With the use of very well characterized chemical conditions (uranium concentration, pH, ionic strength, atmospheric partial pressure), time resolution and then spectral deconvolution, the acquisition of precise emission bands and lifetimes for these different complexes are identified. These spectroscopic fluorescence data are particularly important either for laboratory or for *in-situ* studies [64,65,66]. The other relevant aqueous U(VI) species, (UO_2_)_2_CO_3_^-^(OH)_3_^-^, does not show any fluorescence at room temperature [67]. For comparison, the fluorescence lifetimes and main emission bands of selected aqueous, adsorbed, and solid reference substances are given in Table 2.

#### 2.4.3. U(VI) sorption on mineral surface

TRLFS technique was performed to study the U(VI) sorption and species on mineral surfaces, for example, onto gibbsite [68,69], Al-hydroxide [70], muscovite [71], calcite [72,73,74], silica [22,75], montmorillonite [2] and kaolinite [62]. TRLFS provides information on both fluorescence lifetime and spectral signature of the adsorbed species, which offers access to a number of different species and their spectral identity.

**Table 2:** Fluorescence data of aqueous, adsorbed, and soil reference species.

U(VI) species	Lifetime (µm)	Main emission bands (nm)				Line width (description)	Reference
UO_2_^2+^	0.9-2	488	509	533	559	Narrow	[64,65]
UO_2_(OH)^+^	32.8-80	497	519	544	570	Narrow	[64]
(UO_2_)_2_(OH)_2_^2+^	2.9-9.5	497	519	542	570	Narrow	[65,66]
(UO_2_)_3_(OH)_5_^+^	6.6-23	496	514	535	556	Broad	[64,66]
(UO_2_)_3_(OH)_7_^-^	10-230	503	523	547	574	Broad	[64,66]
UO_2_OSi(OH)_3_^+^	19	500	521	544	570	Broad	[75]
Uranyl hydroxide solids							
Fresh precipitate	63	Not determined				Structureless	[76]
Aged precipitate	131	Not determined				Structureless	
Sorbed U(VI) surface species on montmorillonite							
S1	170-270	500	521	545	570	Broad	[76]
S2	2-12	494	513	533	555	Broad	
S3	15-25	Not determined				Structureless	
S4	80-110	504	525	549	575	Narrow	
Sorbed U(VI) surface species on the edge faces of muscovite							
1	1.15	503	522	545	569	Structureless	[71]
2	9.65	502	522	545	570	Structureless	
schoepite	63-131	Not determined				Structureless	
Sorbed U(VI) surface species on silica							
≡SiO_2_UO_2_	0.17	Not determined				Broad	[22]
≡SiO_2_UO_2_OH^-^	0.36	Not determined				Broad	
Sorbed U(VI) surface species on albite							
τ1	0.49-1.5	500	521	543	573	Structureless	[77]
τ2	10.6-21	500	520	543	570	Broad	

TRLFS studies of adsorbed uranyl invariably indicate the presence of two or more surface species. For example, two uranyl surface species were presented on silica, muscovite, albite and smectites surface [22,71,77,78]. The emission spectra with the short or long fluorescence lifetimes were hypothesized to correspond to an inner-sphere mononuclear complex and polynuclear uranyl surface species, respectively [68,71]. Chisholm-Brause *et al.* [78] used TRLFS to investigate uranyl surface species on smectite edge sites. They reported that at pH 4.1-5.5 only one species was detected, which was interpreted as a monomeric species ≡AlOUO_2_^+^. By raising the pH, a second surface species shifted by 155 cm^-1^ to lower energy values appeared and with increasing the pH gradually up to 8.1 this species became more important. It was interpreted as a hydrolyzed uranyl surface complex ≡SiO(UO_2_)_3_(OH)_5_. Uranyl surface speciation on silica particles was concluded that two fluorescent surface complexes were distinguished by their fluorescence spectra, ≡SiO_2_UO_2_ and ≡SiO_2_UO_2_OH^-^ dominating at pH 5.5 and 7.7, respectively [22]. It was also hypothesized that a third non-fluorescence species, ≡SiO_2_(UO_2_)OHCO_3_^3-^, occurring around pH 8.6. The different species of adsorbed U(VI) can be identified from the TRLFS analysis, which can be used in the model simulation of U(VI) sorption on minerals.

TRLFS studies of uranyl ion adsorbed on montmorillonite showed the presence of four species denoted as S1, S2, S3 and S4 [76]. Species S1 and S2 were assigned to uranyl bound at aluminol sites via inner-sphere complexation and at ion exchange sites, respectively. The featureless spectrum of species S3 correspond to oligomeric uranyl complexes formed at high surface coverage, and species S4 was assigned to an outer-sphere electrostatically bound complex of uranyl. Although the spectrum of species S4 was visually similar to that of free UO_2_^2+^, S4 was associated with UO_2_(OH)^+^ or other hydrolyzed uranyl species. 

It is well known that the concentration of U(VI) is crucial to sorption species. For uranyl sorption onto gibbsite, at high U(VI) concentration, [U] = 1.0 × 10^-5^ mol/L, two species of adsorbed uranyl with widely different fluorescence lifetimes but similar vibronic spectra were discerned at ambient temperature [68]. At [U] = 8.4 × 10^-7^ mol/L, Chang *et al*. [69] found four components denoted as species A, B, C and D. Species A and B are likely to correspond to inner-sphere surface aluminol complexes ≡AlO(UO_2_)^+ ^and ≡AlO(UO_2_)OH^0^, while species C is hypothesized to correspond to electrostatically bound uranyl complexes (predominantly [UO_2_(OH)_3_]^-^), and D is likely to be a precipitate of schoepite.

It has been reported that the spectroscopic data can be used to characterize the calcite-water interface in the presence of U(VI) [72,73,74]. The luminescence data indicate the presence of at least two sorption complexes with changing the proportion of U(VI) loading in air-equilibrated calcite suspensions at pH 8.3 ([U(VI)] < 100 µmol/L). The uranyl triscarbonate complex is dominated at low-surface coverage. And at higher surface loadings, the sorption complex is intermediate between the triscarbonate species and uranyl incorporated into bulk polycrystalline calcite. Combined the EXAFS and luminescence data, it is indicated that the sorption complexes formed at the calcite surface are triscarbonate-like U(VI) complexes, with a change in interaction with the surface as the surface loading increases. The formation of U(VI) hydroxide/carbonato precipitates is observed at high concentrations [U(VI)] > 500 µmol/L. Reeder *et al*. [73,74] have demonstrated that significant amounts of uranyl can be incorporated into calcite and aragonite during laboratory coprecipitation experiments (Ca^2+^, HCO_3_^-^ and U(VI) concentrations were maitained in the range 10-15 mmol/L, 10-15 mmol/L, and 10-82 µmol/L, respectively). They observed a different equatorial coordination of UO_2_^2+^ in calcite, which suggested that the change is required for U(VI) incorporation into calcite [73]. An important observation is that uranyl is incorporated differentially between nonequivalent growth steps on the common (10ī4) face of calcite by combining with XAFS, micro-XAS analysis [74].

#### 2.4.4. Effect of humic acid on uranyl sorption

**Figure 11:**
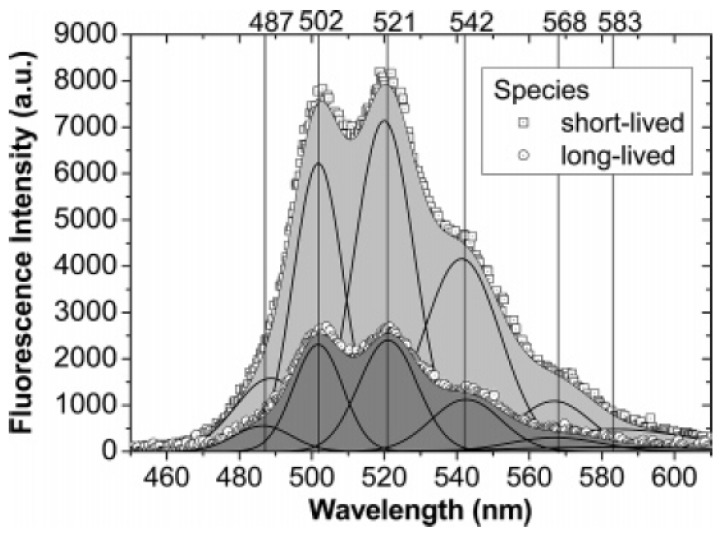
Deconvoluted fluorescence spectra with characteristic positions of the fluorescence emission bands for the UO_2_-kaolinite measured at pH~7 [11].

Krepelova *et al.* [11] applied TRLFS to study the U(VI) surface complexes on kaolinite in the presence or absence of humic acid. An example of U(VI) surface species adsorbed on kaolinite is shown in Figure 11. The deconvoluted fluorescence spectra of a single species with their characteristic emission bands are situated at 486.9 ± 0.9, 501.8 ± 0.6, 520.6 ± 0.9, 541.7 ± 0.7, 567.8 ± 1.5 and 583.3 ± 0.6 nm. The positions of the peak maxima are shifted significantly to higher wavelengths relative to that for the free uranyl ions in perchlorate medium [11]. Two fluorescent surface complexes were distinguished by their fluorescence lifetimes. In the ternary system, U(VI) is not preferred to directly bound on kaolinite surface, but it is adsorbed as a uranyl-humate complex as a “bridge” between U(VI) and kaolinite. The hydration shell of the U(VI) surface complexes is displaced with complexed HA, which is simultaneously distributed between kaolinite particles.

## 3. X-Ray Absorption Fine Structure (XAFS) Spectroscopy

Synchrotron-based X-ray absorption fine structure (XAFS) spectroscopy is an important tool in environmental research for providing unique structural and composition information on surface complexes *in-situ* and under relatively dilute concentrations. XAFS is therefore an element-specific, bulk-sensitive, and nondestructive method. A drawback of XAFS studies is that the metal ion concentrations required usually exceed those expected under natural conditions. The intensities of XAFS spectra of metal ions are dependent on the concentration of metal ions on solid particles or in solution. If the concentration is low, the XAFS spectra of the metal ions generally have very high noise and cannot be used to get local atomic structure information. 

The XAFS technique is an extremely useful speciation method. The application of XAFS has now been largely carried out for understanding the speciation of lanthanide/actinide complexes on mineral surfaces [14,79,80,81]. Atomic distances between the adsorbed metal and sorbent atoms can be obtained by XAFS analysis and the interface reaction mechanisms can be inferred from the sorbate-sorbent polyhedral linkage. XAFS has proven to be useful in discerning sorption mechanisms such as inner-sphere surface complexation [82,83,84,85], outer-sphere surface complexation (physical sorption) [86,87], coprecipitation/structural incorporation [73,74,88] and redox reactions [89,90,91]. 

The outline of XAFS spectroscopy (including a general concept, theory, and data analysis) and an overview of lanthanide/actinide sorption speciation using extended X-ray absorption fine structure (EXAFS) spectroscopy are described in this section. The examples presented in this section are not meant to be an exhaustive treatise on the theme of sorption speciation using EXAFS in the nuclear waste disposal safety issues. Instead, herein we selected the examples which illustrated important aspects of research dealing with sorption speciation. Related work from other research groups are referenced as literature citations.

### 3.1. Outline of XAFS Spectroscopy

X-rays are absorbed by matter primarily through the photoelectric effect, whereby at energies above the ionization threshold electrons from inner core states are excited into empty outer-lying states. XAFS spectra are obtained by measuring the X-ray absorption or fluorescence of a given sample as a function of the wavelength. 

**Table 3:** X-ray K- and L-edge energies of the actinide/lanthanide elements in eV, data are from [92].

Z	Element	E(K)	E(L_I_)	E(L_II_)	E(L_III_)
57	La	38924.6	6266.3	5890.6	5482.7
58	Ce	40443.0	6548.8	6164.2	5723.4
59	Pr	41990.6	6834.8	6440.4	5964.3
60	Nd	43568.9	7126.0	6721.5	6207.9
61	Pm	45184.0	7427.9	7012.8	6459.3
62	Sm	46834.2	7736.8	7311.8	6716.2
63	Eu	48519.0	8052.0	7617.1	6976.9
64	Gd	50239.1	8375.6	7930.3	7242.8
65	Tb	51995.7	8708.0	8251.6	7514.0
66	Dy	53788.5	9045.8	8580.6	7790.1
67	Ho	55617.7	9394.2	8917.8	8071.1
68	Er	57485.5	9751.3	9246.3	8357.9
69	Tm	59389.6	10115.7	9616.9	8648.0
70	Yb	61332.3	10486.4	9978.2	8943.6
71	Lu	63313.8	10870.4	10348.6	9244.1
89	Ac	106755.3	19840.0	19083.2	15871.0
90	Th	109650.9	20472.1	19693.2	16300.3
91	Pa	112601.4	21104.6	20313.7	16733.1
92	U	115606.1	21757.4	20947.6	17166.3
93	Np	118678.0	22426.8	21600.5	17610.0
94	Pu	121818.0	23097.2	22266.2	18056.8
95	Am	125027.0	23772.9	22944.0	18504.1
96	Cm	128200.0	24460.0	23779.0	18930.0
97	Bk	131590.0	25275.0	24385.0	19452.0
98	Cf	135960.0	26110.0	25250.0	19930.0
99	Es	139490.0	26900.0	26020.0	20410.0
100	Fm	143090.0	27700.0	26810.0	20900.0
101	Md	146780.0	28530.0	27610.0	21390.0
102	No	150540.0	29380.0	27610.0	21390.0
103	Lr	154380.0	30240.0	29280.0	22360.0

The spectral scan is performed in the vicinity of an X-ray absorption edge of a chosen target element. Unlike most of spectroscopic methods, however, all heavy metals are spectroscopically active, and their spectral features do not overlap since their K or L edges are separated by several hundreds of electron volts. This method can therefore be used to speciate successively heavy metals in compositionally complex matrices by sequentially tuning to one of their absorption edges [13]. A list of absorption edge energies for the actinide/lanthanide elements is given in Table 3. 

**Figure 12:**
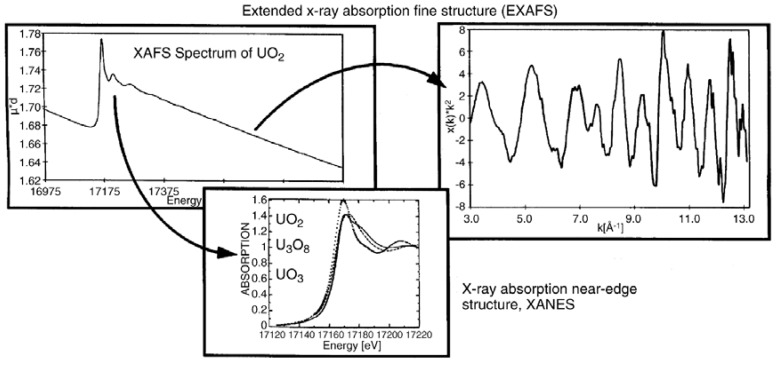
Raw data U L_III_-edge XAFS spectrum of UO_2_(s). The *k*^3^-weighted EXAFS extracted from the raw data (right) and the normalized, background subtracted XANES (bottom), compared to that for U_3_O_8_ and UO_3 _[14].

Figure 12 depicts a typical X-ray absorption spectrum of UO_2_(s), where the product of the absorption coefficient and the sample thickness as a function of photon energy are shown for UO_2_, recorded at the U L_III_-edge. A sharp rise or edge is observed at the absorbing element core state threshold or ionization energy (*E*_0_) which renders this technique element specific. According to the principle quantum number of the electron being excited, these abrupt changes are referred to as *K*, *L*, *M*, *etc.* absorption edges [14]. Detailed description of the physical principle of the method and the data reduction is not attempted herein. Based on the backscatter processes responsible for the oscillatory structure, the photon energy range in an X-ray absorption spectrum is generally divided into two parts: the X-ray absorption near edge structure (XANES) at lower energies (-20−30 eV) and the extended X-ray absorption fine structure (EXAFS) at higher energies (30−1,000 eV). XANES contains chemical and structural information of the metal’s oxidation state, coordination number, and geometry. EXAFS is produced when photoelectrons leaving the absorbing atom(s) are backscattered by the surrounding nearest neighbor atoms. EXAFS data contain information of the absorbed atom’s environment, e.g., coordination number, nearest neighbor, bond distances, and, in ideal cases, bond angles, *etc.* [93]. 

The XANES region involves transitions about *E*_0_, the valence state of the absorbing atom can also be determined from XANES data. The edge shifts towards higher energies in the order UO_2_, U_3_O_8_, UO_3_ due to reduced shielding of the core electrons (*i.e.,* increase in *E*_0_ of the core electrons) associated with the increase in mean valence state across the series (Figure 12). By comparing XANES edge energies of samples with given oxidation states with that of an unknown, the oxidation of the unknown can be identified.

In the EXAFS region, the wave function of the excited photoelectron in the core region is modulated by interference of the outgoing wave function with a fraction, which has been backscattered on the neighboring atoms. In this sense the EXAFS oscillatory pattern is quite literally an inter ferrogram of the atomic arrangement surrounding the absorbing atoms. It therefore contains metrical parameters, such as number and type of neighboring atoms and their distance to the absorbing atom.

### 3.2. Sorption of Radionuclides on Minerals

The actinide elements U, Np, and Pu form oxo-cations (“actinyl-cations”) in oxidizing aqueous environments [88]. The environmental behavior of the actinyl ions U(VI)O_2_^2+^, Np(V)O_2_^+^ and Pu(V,VI)O_2_^(+,2+)^ in the geosphere is to a large extent controlled by sorption reactions with minerals. Many of the published studies have been concerned with actinide/lanthanide coordination environments at the surfaces of clay minerals and metal oxides. The formation of short actinide oxygen bonds in actinyl cations for the (V) and (VI) oxidation states leads to characteristic XANES features, which can be used for their identification. The redox speciation of actinyl cations by XANES was studied by many researchers [89,90,91]. More recent reports aim at understanding actinide/ lanthanide-HA/FA interaction by EXAFS [94,95,96,97]. The importance of such studies rests upon in the ability of HA and FA to form complexes with actinides/lanthanides, which renders them an important role in natural processes determining the mobility and fate of metal cation ions in the environment.

### 3.3. U(VI) L_III_-edge EXAFS Study of Sorption Speciation

The adsorption of the uranyl ion (UO_2_^2+^) in contact with amorphous silica (SiO_2_) [82,83,98], TiO_2_[99], alumina (Al_2_O_3_) [83,100], Fe_2_O_3_[100], montmorillonite [76,83], imogolite [84], kaolinite [11] and calcite [72,73,74] shows that the uranyl ion structure is bounded with two axial oxygen atoms detected at bond distance about 1.8 Å. For the uranyl ion sorption on silica and alumina at low pH, the equatorial oxygens split into two distinct shells with bond distances of ~2.30 Å and ~2.49 Å, no near-neighbor silicon or uranium is detected [82,83]. It has been suggested that the splitting of the O_eq_ shell can be used to infer inner-sphere coordination of U(VI) at water-mineral interfaces. A uranium shell at 4.0 Å is observed in the near-neutral pH (~6.5) samples of uranyl ions on silica and γ-alumina. A silicon shell at 3.10 Å is observed in the sample of uranyl ion sorption on silica at pH 6.5 [83]. EXAFS analysis indicated that adsorption of the uranyl ions onto the silica and γ-alumina surfaces appear an inner-sphere, bidentate complexation mode, and formed polynuclear surface complexes occurring at near-neutral pH. For sorption on the montmorillonite at low pH, a single equatorial oxygen shell is observed at 2.4 Å, with a coordination number of 6 ± 1. At near-neutral pH (6.41) and high ion concentration (0.1 mol/L NaCl), two separate equatorial shells are also observed with bond distances of 2.30 and 2.48 Å [83]. These results suggest that adsorption of the uranyl ion onto montmorillonite at low pH occurs via ion exchange, leaving the inner-sphere uranyl aquo-ion structure intact. At near-neutral pH and in the presence of competing cation ions, inner-sphere surface complexation with the surface predominate uranyl sorption on montmorillonite. EXAFS analysis have shown that uranyl is adsorbed onto silica and γ-alumina as bidentate surface complexes [83], which is in contrast to some interpretations from respective TRLFS results [22,69]. Since detailed structural information on the sorption species cannot be derived by TRLFS, the sorption complexes were obtained by using previously published data of uranyl on mineral, or with the combination surface complexation modelling [2,22].

**Figure 13:**
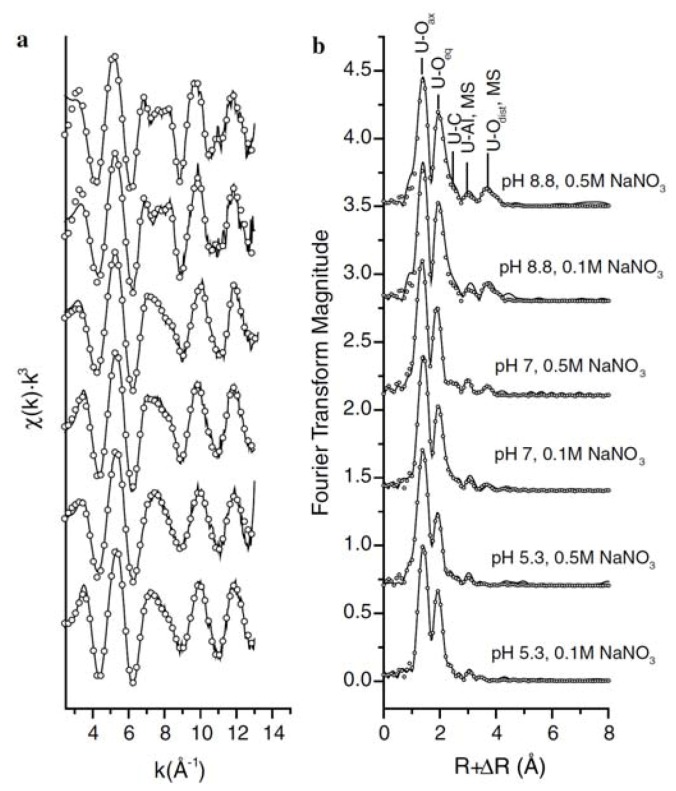
(a) Normalized, background-subtracted k^3^-weighted U L_III_-edge EXAFS spectra of U(VI) adsorbed imogolite samples. (b) Fourier transformed k^3^-weighted U L_III_-edge EXAFS spectra of U(VI) adsorbed imogolite [84].

The structures of U(VI) surface species at the imogolite-water interface has been examined by Arai *et al*. [84]. Figure 13 shows the k^3^-weighted EXAFS spectra of U(VI) adsorbed onto imogolite. The structural parameters of all samples contain two axial oxygen distances at approximately 1.8 Å, and six equatorial oxygen distances at ~2.4 Å, indicating the presence of a O=U=O transdioxo structure. Fourier transformed (FT) peaks at ~2.9, 3.3, and 4.2 Å are attributed to C, Al, and distal O (in carbonate groups) shells. They did not observed any obvious splitting of U-O_eq_ shells in the FTs, and the presence of Al backscatters that can suggest the presence of inner-sphere surface species. Principal component analyses (PCA) were conducted to elucidate the number of significant surface species. The results suggested that at pH 8.8, bis-carbonato inner-sphere and tris-carbonato outer-sphere surface species are present. At pH 7, bis- and non-carbonato inner-sphere surface species co-exist, and the fraction of bis-carbonato species increases slightly with increasing ionic strength. At pH 5.3, U(VI) non-carbonato bidentate mononuclear surface species predominate.

EXAFS studies on experimentally precipitated U(VI) containing calcite indicate that UO_2_^2+^ becomes incorporated. The uranyl ion retains its two axial oxygen (O_ax_) neighbors but exhibits a carbonate coordination which is different from the Ca^2+^-CO_3_^2-^ coordination in calcite [73,74]. In contrast to aqueous carbonato complexes, five equatorial oxygens (O_eq_) at a bond distance of 2.33-2.36 Å and a splitted carbon coordination sphere with carbon atoms at 2.9 and 3.2 Å have been found. This indicates a combination of monodentate and bidentate bound carbonate groups with significant static disorder, as indicated by their Debye-Waller factors.

Křepelová *et al*. [94] investigated the influence of HA on the near-neighbor surrounding of U(VI) in kaolinite surface complexes by U L_III_-edge EXAFS. In the binary U(VI)-kaolinite system, uranium forms inner-sphere surface complexes by sharing edge with aluminum octahedra and/or silicon tetrahedra. In the ternary system, EXAFS data were obtained by including two uranium coordination shells with two axial (O_ax_) and five equatorial (O_eq_) oxygen atoms at bond distances of 1*.*77 ± 0*.*02 and 2*.*34 ± 0*.*02 Å, respectively, and two coordination shells with one Al/Si atom at 3.1 and 3.3 Å. The results suggested that HA and atmospheric CO_2_ as well as pH had no influence on the EXAFS structural parameters in the pH range of 5-8. They concluded U(VI) preferred to adsorb directly onto kaolinite and not to HA that was bound to the clay surface. 

In the polarized extended X-ray absorption fine structure (P-EXAFS) spectroscopy, neighboring atoms along the polarization direction of the X-ray beam are preferentially probed, and atoms located in a plane perpendicular to this direction are attenuated. P-EXAFS has been successfully used to determine the uptake processes of metal ions on clays. Using P-EXAFS, Dähn *et al*. [101] investigated the uptake of Th(IV) on montmorillonite. Auwer *et a*l. [99] addressed the orientation and structure of the uranyl oxocation complex onto the TiO_2_ surface. Their polarized XANES measurements show that the uranyl rod sorbs nearly parallel to the rutile surface. Both grazing incidence EXAFS on the (110) plane and isotropic EXAFS on polycrystalline TiO_2_ reveal comparable sorption behavior: on average, the uranyl oxocation bonds to the surface as a bidentate complex with two short oxygen distances at 2.32 Å and three larger distances at 2.47 Å. Grazing incidence EXAFS on the (001) plane shows an unexpected low signal to noise ratio due to the lower uranium uptake. Data analysis suggests the formation of outer-sphere uranium surface complexes on this plane.

### 3.4. Np(IV/V) L_III_-edge EXAFS Study

Many long-lived radionuclides of environmental importance have access to more than one oxidation state in environmental systems. The different oxidation states have very different geochemical properties. Likewise, Np may occur as Np(IV) or NpO_2_^+^. The elements in different oxidation state may be reflected in changes in K_d_ (distribution coefficient) of several orders of magnitude [102]. 

Np(V) sorbs onto a variety of synthetic and natural minerals with decreasing affinity in the sequence calcite > goethite >> MnO_2_ ≈ clays [103]. Many EXAFS studies showed that the mineral chemisorbed the neptunyl species by inner-sphere surface complexation. Differences of pentavalent neptunyl species in the structural environment are found by the investigation of the neptunyl adsorbed onto mineral and neptunyl in the supernatant [88,103]. EXAFS data shows differences in the Np(V)-O_ax_ bond distance of 1.85 ± 0.01 Å for the adsorbed Np-calcite, Np-geothite, and 1.82 ± 0.01 Å for the solution species (Table 4). The equatorial environment of the neptunyl in solution and adsorbed neptunyl shows about five oxygen neighbors at 2.45~2.51 Å. Heberling *et al*. [88] detected an additional feature in the adsorbed species R-space spectrum, which was related to carbonate neighbors, three to six carbon backscatters (C_eq_) at 3.05 ± 0.03 Å and 3 to 6 oxygen backscatters (O_eq2_) at 3.31 ± 0.02 Å. The differences in the Np(V)-O_ax_ bond distance and the C_eq_ and O_eq2_ backscatters which are only present for the adsorbed species indicate inner-sphere bonding of the adsorbed neptunyl species to the calcite surface. Combes *et al*. [103] identified the Np-Fe second-neighbor pair correlation and suggested that Np(V) sorbs at the goethite water interface as an inner-sphere complex.

**Table 4:** Results of EXAFS data obtained for Np(V) aquo ions, Np(V) humate and Np(V) adsorbed to α-FeOOH and calcite.

System	Np-O_ax_		Np-O_eq_		Reference
	R (Å)	N	R (Å)	N	
aquo ion	1.82 ± 0.01	2.5 ± 0.6	2.45 ± 0.02	4.8 ± 1.0	[88]
	1.83	1.6	2.52	5.2	[103]
NpO_2_(CO_3_)^-^	1.82 ± 0.02	2	2.49±0.03	4~5	[104]
Np-humate	1.83	2	2.47	5.3	[105]
Np-geothite	1.85	2.2	2.51	5.5	[103]
Np-calcite	1.85 ± 0.01	2.1 ± 0.2	2.46 ± 0.01	5.1 ± 0.6	[88]

By applying chemically modified HAs with blocked phenolic/acidic -OH groups, the coordination environment of neptunium(V) in complexes with humic substances are studied. The Np(V)-humic complex is observed to have two distinct nearest neighbor oxygen distances, axial Np-O bond distances of 1.84-1.85 Å and equatorial plane 2.48-2.49 Å. The comparison of the structure parameters of the Np(V) humates with those of Np(V)-Bio-Rex70 also point to the fact that at pH 7 carboxyl groups dominate the interaction between Np(V) and HA. No Np-Np backscatter peak is observed in the EXAFS spectra [105]. The interaction between Np(V) and HSs is dominated by predominant monodenated carboxylate groups. Similar results were also observed for Np(IV) complexation by humic substances by Schmeide *et al*. [106], and they reported that Np^4+^ is surrounded by about 10 oxygen atoms at an average bond distance of 2.36 ± 0.02 Å and the carboxylic groups are the main complexing site of humic substances responsible for binding neptunium(IV) in pH 1 solutions. 

### 3.5. Others

The aquatic chemistry of trivalent 4f- and 5f-elements has been demonstrated to be very similar. Geckeis and Rabung [49,59] compared EXAFS data for Am(III), Gd(III) and Lu(III), and they suggested that the coordination numbers and M-O (metal-oxygen) bond distances were not significantly different from those of the respective aquo ions (Table 5). 

**Table 5:** Results of EXAFS data obtained for lanthanides/actinides adsorbed to γ-Al_2_O_3_ and clay minerals.

System	Binding	R (Å)	N	Reference
Am-aquo ion	Am-O	2.473(2)	8.3 ± (10-20%)	[107]
Am/Cm-aquo ion	Am/Cm-O	2.48 ± 0.002	10.3 ± 0.33	[108]
		2.45 ± 0.002	10.2 ± 0.33	
Gd-aquo ion	Gd-O	2.42	9-8	[109]
Lu-aquo ion	Lu-O	2.31	9.1 ± 0.3	[110]
Am-kaolinite, pH6-8	Am-O	2.481(8)	8.9(-6.2) ± (10-20%)	[107]
Am-smectite, pH6-8	Am-O	2.474(7)	8.5(-7.2) ± (10-20%)	[107]
Am-marl colloids	Am-O	2.50 ± 0.02	10.3 ± 1.3	[111]
Gd-γ-Al_2_O_3_	Gd-O	2.45 ± 0.02	8.7 ± 1.5	[49]
Lu-γ-Al_2_O_3_	Lu-O	2.28 ± 0.02	7 ± 1.5	[49]

However, asymmetric peaks and large Debye-Waller factors indicate the presence of slight variations in M-O bond distances of the surface bound trivalent metal ions (Gd, Am, Lu), which cannot be resolved in the spectra. 

**Figure 14:**
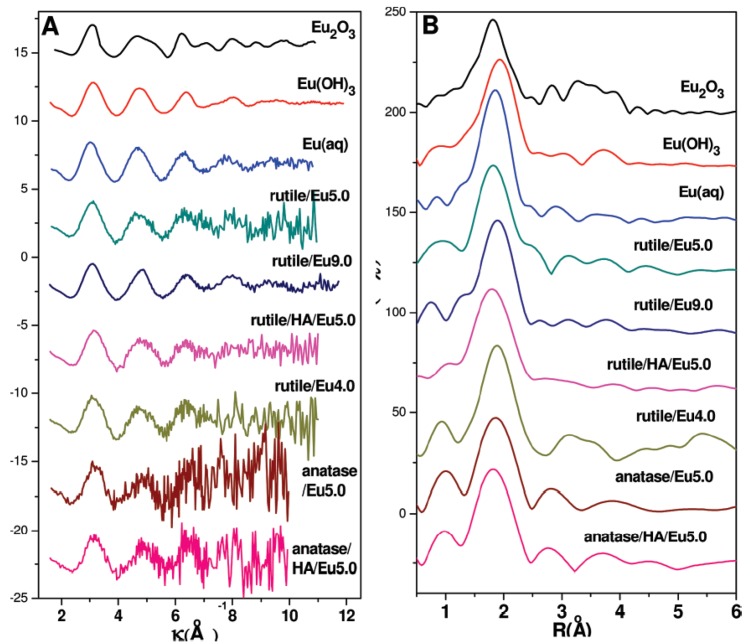
*k*^2^-weighted EXAFS spectra (A) and the corresponding Fourier transforms (B) of reference samples and sorption samples. Eu(III) adsorbed on rutile at pH 5.0 (rutile/Eu5.0), at pH 4.0 (rutile/Eu4.0) and at pH 9.0 (rutile/Eu9.0). The presence of HA at pH 5.0 (rutile/HA/Eu5.0 and anatase/HA/Eu5.0).Eu(III) adsorbed on anatase at pH 5.0 (anatase/Eu5.0). C(TiO_2_) = 0.3 g/L, C(Eu(III))_(initial) _= 3.0 × 10^-5^ mol/L, C(NaClO_4_) = 0.01 mol/L, C(HA)_(initial) _= 7.5 mg/L, pH = 4.0 ± 0.1 or 5.0 ± 1, T = 25 ± 1 ^o^C [97].

In our research group, we have focused on studying the structure information of trivalent Eu(III) ions at atomic molecular level on the oxides and mineral surfaces [7,31,97,112,113] by using Eu L_III_-edge EXAFS. It was found that Eu(III) was bound to about seven or eight O atoms at a bond distance of about 2.40 Å, which is different from the aquo complexes and thus indicative for inner-sphere surface complexation on oxides or mineral surfaces. The presence of natural organic matter has significant influence on the species and microstructures of Eu(III) adsorbed on solid particles, and the influence of addition sequences of HA on Eu(III) sorption to attapulgite is different [31]. The species and local atomic structures of Eu(III) on oxide or mineral surfaces are not only affected by pH values, but also by the presence of humic substances. The *k*^2^-weighted EXAFS spectra and the corresponding Fourier transforms of Eu(III) adsorbed on anatase and rutile at different experimental conditions are shown in Figure 14. 

EXAFS data for the Eu(III) sorption samples and Eu(OH)_3_(s) are obviously different. The attempts to include Eu-Eu shell contribution to the EXAFS spectroscopy data suggest the formation of surface complexes including a polymeric form of Eu at low pH, while compelling formation of precipitation on solid surface at high pH values. The EXAFS analysis show that Eu(III) is adsorbed on TiO_2_ as inner-sphere surface complexes, showing little change in the pH-dependent sorption of Eu(III) as a function of ionic strength.

**Figure 15:**
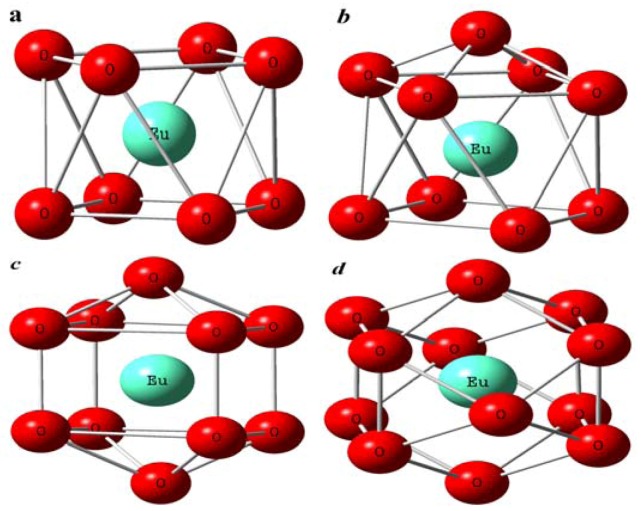
The different imaginary Eu(III) structures according to EXAFS results of the first order coordination shells. (a): dodecahedron; (b): mono-crown anti-tetragonal-prism; (c): bi-crown-dodecahedron; (d): icosahedron [31].

The Eu-O bond distance and coordination number for Eu(III) on attapulgite in the presence of HA are quite different for different addition sequences of Eu(III) and HA [31]. The difference suggests that the configuration of Eu(III) is different (Figure 15) for different sorption sequences, which indicates that the mechanism and species of Eu(III) sorption to attapulgite has been changed in the different addition sequences. The EXAFS analysis indicates that the influence of addition sequence of HA/Eu(III) on Eu(III) sorption to attapulgite is quite different, and the species are dependent on the different complexation sequences. Although the differences in the amount of Eu(III) adsorbed on solid particle is not found for the different addition sequences by batch techniques, the species and local atomic structures of Eu(III) on solid particles are really affected by different complexation sequences. 

## 4. Density Functional Theory (DFT)

The availability of faster computers and the great advances of theoretical methods over the last thirty years have permitted systems with increasing complexity to be calculated with remarkable accuracy [17,114]. Theoretical calculations are becoming routinely used as an indispensable tool in chemical research. Density functional theory (DFT) is one of the most widely used methods for “*ab initio*” calculations of the structure of atoms, molecules, crystals, surfaces, and their interactions. DFT calculations yield structural, force, and total energy information, as well as the system charge density. These properties enable comparison and connection with experiment and identification of underlying mechanisms through analysis of the electronic structure. The description of the conceptual, fundamental and performance aspects of DFT is not attempted herein, and relative information is available in literature [115]. 

DFT calculations have been used to study metal-ligand interactions in organometallic and coordination compounds and to study the adsorption process on metals and on oxide surfaces with remarkable success [17,116,117,118,119,120,121]. In theoretical studies of ion adsorption onto mineral surfaces, cluster models were exploited by previous workers based on the fact that the adsorption process is a local phenomenon [118,119,120,121]. This theoretical modeling allowed one to get information as nature of the sorption sites and relative energy stabilities of the different surface complexes. These results were directly correlated to the experimental ones in order to better understand sorption processes of radionuclides on mineral surfaces.

What the reader should note in this study is that it belongs to a momentary noticeable trend in the EXAFS community of combining EXAFS analysis with theoretical, either quantum chemical or molecular dynamic, calculations. The combination of theoretical calculations with EXAFS has two main advantages. First, calculated complexes can be verified by comparing the obtained radial distances and coordination numbers with those measured by EXAFS. Second, considering the fact that energy comparison between optimized structures is still less accurate, especially for surface reactions, EXAFS can provide structural constraints for the quantum chemical calculations. Only a few works have demonstrated up to now the potential of this combination of methods for elucidating the structures of surface complexes [119,120,121].

Quite a few quantum chemical calculations complementing experimental data on actinide coordinated complexes in mineral surfaces have been published in recent years, almost all the approximate theoretical models have been applied to uranyl ions chemisorption [118,120,121,122,123,124,125,126,127]. Some works were performed with respect to uranyl surface complexation on minerals and combined with EXAFS data to validate the surface complex structure [120]. Bidentate uranyl complexes could form on edge and corner surfaces of TiO_2_, α-FeOOH and Al(OH)_3_, allowing explanation of the retention properties of minerals. Moskaleva *et al*. [122] examined uranyl adsorption onto the hydrated α-Al_2_O_3 _(001) surface. An outer-sphere adsorption mechanism seemed to be favored on the fully protonated surface. Wheaton *et al*. [123] reported the optimized geometries of various uranyl silicate complexes to understand the ways of uranyl ion bound to a silica interface or a colloid. They assigned the structure at low U loading to the bridged uranyl monosilicate structure, while at high loading the structure corresponds to the uranyl disilicate. Molecular dynamics studies were also carried out to describe uranyl’s interaction with quartz (010) [124]. Both inner-sphere and outer-sphere surface complexes were detected, depending on the protonation state of the surface. 

**Figure 16:**
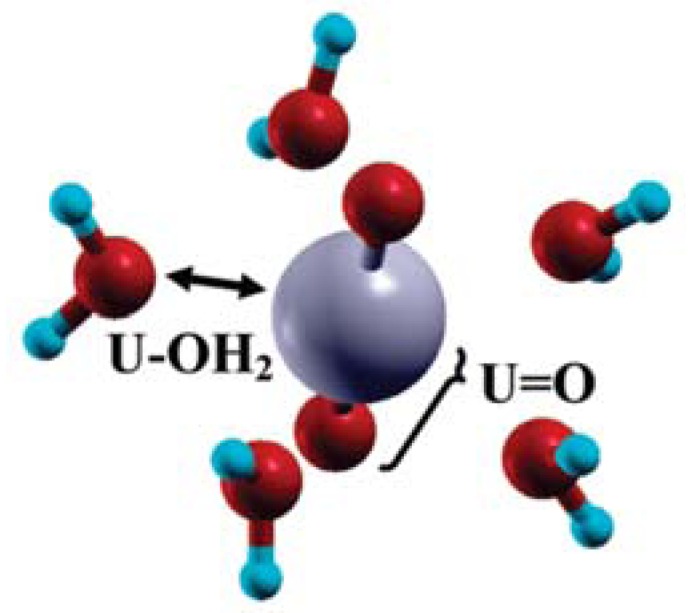
Optimized structure of the pentahydrated uranyl ion, [UO_2_(H_2_O)_5_]^2+^: uranium atom in gray, O atoms in red, and hydrogen ones in blue [125].

From the aqueous uranium speciation diagram, the uranyl ion (UO_2_^2+^) is the main species at pH < 4 [121]. Knowing that the free uranyl ion in acidic solution has five water molecules in its first hydration shell ([UO_2_(H_2_O)_5_]^2+^) [99], DFT calculations were performed on the hydrated uranyl ion with several numbers of water molecules (up to six). Several structures with water molecules in the first and in the second hydration shells were considered. The optimized bond distances for pentahydrared system (Figure 16) were found to be the most stable structure, which are in good agreement with EXAFS data giving 1.77 ± 0.02 Å for U=O and 2.42 ± 0.02 Å for U-O_water_ [99]. 

**Figure 17:**
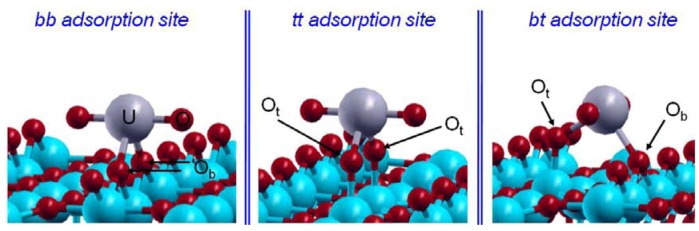
The three crystallographic possible bidentate sorption sites for the uranyl ion on the rutile TiO_2 _(110) face: bridging-bridging (noted *bb*), terminal-terminal (noted *tt*) and finally bridging-terminal (noted *bt*) [127].

Researchers have presented the local atomic structure of the uranyl ion adsorbed onto the rutile TiO_2_ (110) face by DFT calculations. A five layer slab with the atomic positions of its most internal layer frozen to bulk positions was used to study the interaction of uranyl sorption species with the TiO_2 _(110) surface [118]. The uranyl ion is adsorbed as a bidentate complex with an inner-sphere mechanism onto the TiO_2_ rutile (110) face with three water molecules to saturate its first hydration shell [125]. Three possible adsorption sites: one uranyl ion linked to two bridging oxygen atoms, one adsorbed uranyl ion to a bridging oxygen atoms and a terminal one and one adsorbed uranyl ion on two terminal oxygen atoms were performed to the model of the adsorption process (Figure 17). Regarding the relative sorption energies of the uranyl ion, it appeared that the first two structures were the most stable ones. The third structure was less stable than the second one, which agreed with the previous experimental results: only two uranyl surface complexes were observed [118,126]. Perron *et al*. [125] reported the amount of uranyl ion adsorbed on rutile increases while the two structures evolved differently: initially characterized as the most stable one under strongly acidic conditions, the *bb* structure becomes less stable than the *bt* one.

**Figure 18:**
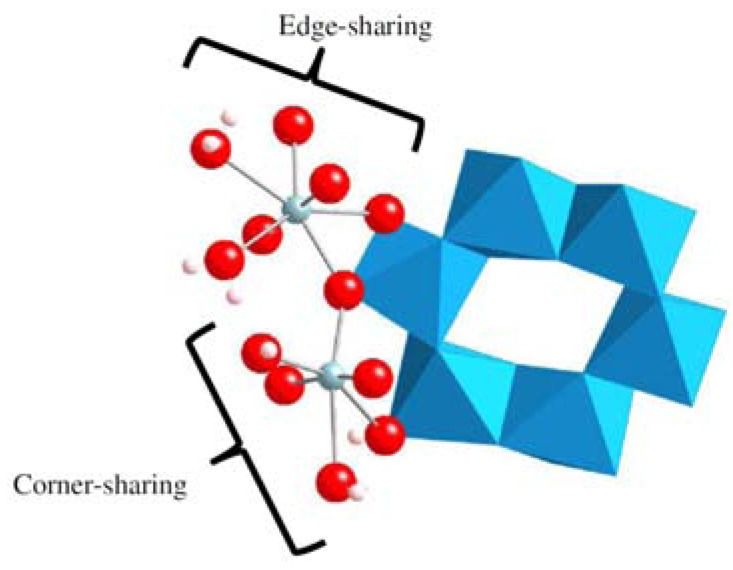
The optimized structure of the corner- and the edge-sharing configuration of uranyl adsorption complexes on gibbsite [121].

Hattori *et al*. [121] investigated the structure of uranyl sorption complexes onto gibbsite (pH 5.6−9.7) by DFT calculations and EXAFS spectroscopy at the U L_III_-edge. They tested two Al (hydr) oxide clusters, a dimer and a hexamer. It was found that the hexamer cluster is more rigid and therefore more appropriate as model for the gibbsite surface. The DFT calculations of (monomeric) uranyl sorption complexes show an energetic preference for the corner-sharing *versus* the edge-sharing configuration on gibbsite edges. They concluded that corner- and edge-sharing surface complexes coexist in adjacent adsorption sites of gibbsite at acidic pH (Figure 18), while in alkaline pH region the dimeric uranyl unit adsorbed onto gibbsite show a corner-sharing configuration. At low pH = 3 or 4, the gibbsite edge faces were strongly positively charged while the basal charges may be neglected, and that uranyl ion was expected to adsorb onto the basal faces. Therefore, surface oxygen atoms bearing in-plane hydrogen atoms were good candidate to link with the uranium atom [127]. From the DFT calculation, even if several complex structures had to be considered previously, all of them gave very similar final complex structures. It was determined only one type of complex structure (a bidentate uranyl complex formation) to be favorable on the (001) gibbsite surface which agrees well with experimental results.

## 5. Conclusions and Future Work

As a conclusion let us reiterate the information content of this review. The examples of lanthanide/actinide speciation studied using TRLFS and EXAFS mentioned herein cover a broad range of topics in actinide/lanthanide science dealing with nuclear waste disposal. Examples of basic research topics in this field including aqueous actinide chemistry, colloid mediated transport of actinides/lanthanides, immobilization or retardation of actinide/lanthanide transport via sorption or migration onto mineral surfaces have been described. Embedded within these examples are some basic as well as advanced aspects of these techniques. In this review we have also attempted to demonstrate the strength of theoretical calculations. DFT combining XAFS leads to corroboration of results but can also be used for refining the modeling of data or identification of systems with actinides in a number of different environments.

As a final word of caution we would like to point out in conclusion the challenges facing experimentalists in studies of actinide/lanthanide speciation in repository far-field scenarios. Such scenarios necessarily deal with dilute systems in a heterogeneous environment. In these cases, it is difficult to obtain data with good signal-to-noise levels. To further complicate matters, the actinides of interest are often present as a mixture of species in a heterogeneous environment. Because of both of these effects, one can often only obtain information concerning the first coordination sphere in such systems. This limits possible interpretation of the data. New applications such as low temperature measurements, spectroscopy of interactions with organic ligands as well as the possibility to detect short living fluorescent actinide species (such as Pu(IV), U(IV) and Am(III)) are expressions of great interest in actinide chemistry at very low concentrations and in direct speciation techniques. We hope to direct theoretical determination of lanthanides/actinides-mineral interfaces, eventually casting results into phenomenological models with predictive capabilities.

The theoretical models and spectroscopy technique studies of the physicochemical properties of lanthanides and actinides in the natural environment are quite important to understand their behavior and interactions with clay minerals or other kinds of sorbents. The batch technique studies of lanthanide/actinide sorption on clay minerals are also crucial to understand the properties of lanthanides/actinides in the environments. The combination of batch technique, theoretical model and spectroscopy analysis at molecular level is necessary to understand the physicochemical behavior of lanthanides/actinides in the natural environment. To understand the physicochemical behavior of lanthanides/actinides in the natural environment, it is necessary for us to understand the species and the structures of lanthanides and actinides at molecular level, and also the information about the sorption, diffusion, migration and desorption properties of lanthanides/actinides at solid-water interfaces. These informations can not be achieved from one of the methods or techniques mentioned above. The combination of batch experiments at macromolecular level, the spectroscopy analysis at molecular lever, modeling the experimental data with the parameters derived from batch and spectroscopy analysis, and theoretical simulation etc are crucial in future work. 
